# Complete genome sequences of 11 *Streptococcus pneumoniae* strains isolated from acute infectious purpura fulminans, sepsis, and pneumonia

**DOI:** 10.1128/mra.00773-23

**Published:** 2023-12-22

**Authors:** Takane Kikuchi-Ueda, Shintaro Maeno, Yasuhiro Gotoh, Yoshitoshi Ogura, Ryuichi Fujisaki, Tetsuya Hayashi

**Affiliations:** 1 Department of Microbiology and Immunology, Teikyo University School of Medicine, Itabashi, Tokyo, Japan; 2 Department of Bacteriology, Faculty of Medical Sciences, Kyushu University, Fukuoka, Japan; 3 Division of Microbiology, Department of Infectious Medicine, Kurume University School of Medicine, Kurume, Fukuoka, Japan; 4 Department of Sport and Medical Science, Faculty of Medical Technology, Teikyo University, Tokyo, Japan; University of Maryland School of Medicine, Baltimore, Maryland, USA

**Keywords:** purpura fulminans, *Streptococcus pneumoniae*, complete genome sequences

## Abstract

The complete genome sequences of 11 Japanese *Streptococcus pneumoniae* isolates were determined by hybrid assembly of long and short reads, including two strains isolated from patients with acute infectious purpura fulminans, six strains from patients with sepsis, and three strains from patients with pneumonia.

## ANNOUNCEMENT

*Streptococcus pneumoniae* is one of the important human pathogens responsible for respiratory infections, sepsis, and meningitis. Its infection is occasionally associated with acute infectious purpura fulminans (AIPF) (1, 2). Here, we report the complete genome sequences of 11 *S*. *pneumoniae* strains isolated at the same hospital in Tokyo, Japan, between 2009 and 2018. Of these, two (PF1 and PF2) were blood isolates from patients who developed AIPF, six (Sep1–Sep6) were blood isolates from sepsis patients, and three (Pne2–Pne4) were isolated from the sputa of individuals suffering from pneumonia (Table 1). These strains were isolated from clinical specimens by culturing at 37°C under aerobic condition on blood agar plates (BD Difco).

**TABLE 1:** Strain information and the summary of assembly data^*a*^

Strain	Isolated date	Source	Symptoms	ST	Serotype	Chromosome length (bp)	GC%	No. of CDSs	No. of rRNAs /tRNAs	No. of prophage regions	Illumina sequencing depth	No. of ONT reads	N50 of ONT reads	Accession no. of sequence reads	Accession no. of assembled sequences
PF1	2011/4/24	Blood	AIFP	3787	6A/6B	2,054,748	39.8	2,048	12/58	2	123	288,834	13,416	DRR465246, DRR465247	AP028601
PF2	2009/11/24	Blood	AIFP	558	35B/35D	2,050,376	39.8	2,026	12/58	3	118	152,990	16,596	DRR465248, DRR465249	AP028602
Pne2	2018/5/19	Sputum	Pneumonia	5242	23A	2,194,831	39.6	2,232	12/59	2	129	273,531	8,586	DRR465250, DRR465251	AP028603
Pne3	2018/8/28	Sputum	Pneumonia	5242	23A	2,194,794	39.6	2,235	12/59	2	145	578,953	15,740	DRR465252, DRR465253	AP028604
Pne4	2018/8/31	Sputum	Pneumonia	433	22F	2,066,045	39.7	2,062	12/58	1	131	685,633	10,272	DRR465254, DRR465255	AP028605
Sep1	2012/2/13	Blood	Sepsis	191	7F/7A	2,105,501	39.8	2,090	12/58	1	122	563,045	11,490	DRR465256, DRR465257	AP028606
Sep2	2012/3/25	Blood	Sepsis	343	14	2,143,010	39.7	2,158	12/59	3	121	625,208	15,165	DRR465258, DRR465259	AP028607
Sep3	2012/4/6	Blood	Sepsis	191	7F/7A	2,039,063	39.9	2,021	12/58	4	133	673,905	9,751	DRR465260, DRR465261	AP028608
Sep4	2012/5/10	Blood	Sepsis	5236	10A	2,118,406	39.6	2,103	12/59	4	123	916,487	3,271	DRR465262, DRR465263	AP028609
Sep5	2012/11/29	Blood	Sepsis	433	22F	2,066,045	39.7	2,064	12/58	1	117	450,320	12,388	DRR465264, DRR465265	AP028610
Sep6	2012/5/10	Blood	Sepsis	246	4	2,243,694	39.5	2,218	12/59	1	108	521,908	10,187	DRR465266, DRR465267	AP028611
															AP028612 ^*b*^

^*a*^ AIFP, acute infectious purpura fulminans.

^*b*^ Accession no. of plasmid.

Genomic DNA was extracted from these strains cultured in Todd-Hewitt Broth (BD Difco) at 37°C for 8 h using Genomic-tip (Qiagen). Short-read sequencing libraries were prepared using the NEBNext Ultra II Library Prep Kit for Illumina (New England Biolabs) and sequenced on the MiSeq platform (Illumina) to generate paired-end sequences (301 bp × 2). The Illumina reads underwent trimming and quality control using Platanus_trim v1.0.7 (3). Long-read sequencing libraries were prepared using Rapid Barcoding Kit [Oxford Nanopore Technologies (ONT)] and sequenced using MinION R9.4.1 flow cells (ONT), followed by base calling using Guppy GPU v3.4.4 with the default model (4) and trimming and quality control using NanoFilt v1.0.7 with parameter “--headcrop 100 --min 10k --q 10” (5). Due to the excessive read depth obtained from ONT sequencing, which hindered long-read assembly, the ONT reads were randomly sampled to a depth of ×50 using SeqKit v2.3.0 (6) before being used for hybrid assembly by MicroPIPE (7). The resulting sequences were further polished using Polypolish v0.5.0 with short reads (8) and circularized using Flye (9). DFAST v1.2.6 (10) was used for gene annotation and genome rotation to start with *dnaA*. Sequence types (STs) and serotypes were determined by pubMLST (11) and PneumoKITy v1.0 (12). Prophage regions were predicted using PHASTER (13). All tools were used with default settings.

The chromosome lengths of the 11 strains ranged from 2,039,063 bp to 2,194,831 bp (Table 1). PHASTER analysis predicted the presence of one to four prophage regions, including “incomplete” and “questionable” regions, in each chromosome. Only Sep6 possessed a plasmid (3,133 bp). Among the 11 strains, two pairs exhibited the same ST and serotype. Specifically, the two ST433/22F strains isolated in 2012 and 2018 displayed a difference of merely two single-nucleotide polymorphisms. On the other hand, the two strains obtained from AIFP patients belonged to different STs and serotypes. As the genome sequence for AIFP-associated *S. pneumoniae* is available only for a serotype 19A strain (accession no. LKAA00000000.1) (14), the sequences of AIPF strains obtained in this study will serve as valuable resources for future researches on *S. pneumoniae*.

This study was exempted from ethical review and approval by the Teikyo University Ethical Review Board.

## Data Availability

The sequences obtained in this study have been deposited in the GenBank/EMBL/DDBJ database under the BioProject accession number PRJDB15255 (see Table 1 for details).
